# Epigenetic modifications of chronic hypoxia-mediated neurodegeneration in Alzheimer’s disease

**DOI:** 10.1186/2047-9158-3-7

**Published:** 2014-03-20

**Authors:** Hui Liu, Weidong Le

**Affiliations:** 1 Institute of Neurology, Ruijin Hospital, Shanghai Jiao Tong University School of Medicine, Shanghai 200025, PR China; 2 1st Affiliated Hospital, Dalian Medical University, Dalian 116011, PR China

**Keywords:** Alzheimer’s disease, Chronic hypoxia, Epigenetic modification, DNA methylation, Histone acetylation

## Abstract

Alzheimer’s disease (AD) is the most common neurodegenerative disorder affecting the elderly people. AD is characterized by progressive and gradual decline in cognitive function and memory loss. While familial early-onset AD is usually associated with gene mutations, the etiology of sporadic late-onset form of AD is largely unknown. It has been reported that environmental factors and epigenetic alterations significantly contribute to the process of AD. Our previous studies have documented that chronic hypoxia is one of the environmental factors that may trigger the AD development and aggravate the disease progression. In this review, we will summarize the pathological effects of chronic hypoxia on the onset and development of AD and put forward the possible molecule mechanisms underlying the chronic hypoxia mediated AD pathogenesis. Finally, we propose that epigenetic regulations may represent new opportunity for the therapeutic intervention of this disease.

## Introduction

Alzheimer’s disease (AD) is the most common neurodegenerative disorder in the elderly people. The latest epidemiology study of AD reported that the number of people with the disease in China is significantly increased from 1.93 million in 1990 to 5.69 million in 2010 [1], representing a major health problem and a heavy financial burden on individuals and society as a whole. Clinically, AD is characterized by progressive and gradual decline in cognitive function, accompanying with severe memory loss and, ultimately, decreasing physical functions and death. The clinical manifestations of AD are associated with the specific pathological changes in the brain of the patient. The pathological hallmarks of the disease are extracellular neuritic plaques, intracellular neurofibrillary tangles (NFTs), synaptic loss and neuron degeneration. Aberrant hyperphosphorylation of tau protein is the main constituent of NFTs. Neuritic plaques are composed of abnormal cleavaged β-amyloid (Aβ) peptide and are surrounded by reactive astrocytes and activated microglia [2,3].

AD can be classified into two forms: early-onset familial AD (FAD) and sporadic late-onset form of AD (LOAD). Most cases of AD are sporadic, with only 5% of the total number of AD exhibiting a clear genetic inheritance [4]. Gene mutations in the amyloid precursor protein (APP), presenilin1 (PS1) and presenilin2 (PS2) have been documented as causatives for FAD, and the Apolipoprotein E4 (ApoE4) allele has been associated with LOAD [5]. Several new genetic findings derived from genome-wide association studies (GWAS) have been identified as new susceptibility loci. These include clusterin (CLU), phosphatidylinositol binding clathrin assembly protein (PICALM), complement receptor 1 (CR1), bridging integrator (BIN1), microtubule affinity-regulating kinase4 (MARK4), the ATP cassette transporter (ABCA7), a membrane-spanning 4-domains, subfamily A (MS4A6A), CD33, a CD2-associated protein (CD2AP), and ephrin A1 (EPHA1) [6,7]. While gene mutations undoubtedly play an important role in the etiology of AD, more and more studies support that the environmental risk factors contribute greatly to the disease onset and progression [8-10]. It is believed that most cases of AD arise through interaction between genetic and environmental factors. Gene-environment interaction refers to a certain environmental exposure in the presence of a susceptibility gene [8]. It is thought that various environmental exposures can contribute to the risk of AD, such as aging, diet and nutrition, educational level, exposure to metals, pesticides, diabetes, brain trauma and etc. [11-14]. Among all the environmental exposures, chronic hypoxia has been extensively studied recently [15]. It has been shown that chronic hypoxia may trigger the AD development and may also aggravate the disease progression [15]. Hereby we summarize current findings of the pathological effects of chronic hypoxia on the onset and development of AD and put forward the potential molecule mechanisms of epigenetic modification underlying the hypoxia mediated AD pathogenesis.

### Chronic hypoxia is a risk factor for dementia and Alzheimer’s disease

Chronic hypoxia is a reduction of oxygen supply, which is a direct consequence of hypoperfusion and restricts the function of organs, tissues or cells. Efficient oxygen delivery to brain tissues is essential for normal brain function and cells survival. Clinically, chronic hypoxia is a common pathophysiological event and contributes significantly to the progression of widespread diseases including fever, chronic obstructive pulmonary disease, obstructive sleep apnea syndrome, stroke, cancer, neurodegenerative disorders and etc. [16]. It has been shown that individuals who have suffered severe hypoxia or ischemia are more susceptible to developing AD [17-19]. Researchers reported that Aβ deposited in the brain after severe head injury [12,20], and cardiac arrest induced a time-dependent increase level of Aβ in the serum of heart attack patients [21]. A recent clinical prospective study by Yaffe and colleagues, demonstrated that older women with sleep-disordered breathing and hypoxia had a significantly increased risk of developing cognitive impairment and dementia [22]. Their findings suggested that chronic hypoxia but not sleep fragmentation or duration was associated with the higher risk of mild cognitive impairment or dementia. Another prospective study named Health, Aging, and Body Composition (Health ABC) that took a follow-up observation over the 11 years has shown that anemia was associated with an increased risk of developing dementia among older adults [23].

Consistent results have been published in transgenic mouse models or cell models showing the impact of chronic hypoxia on AD. It is found that hypoxia increased both mRNA and protein levels of APP in cells and elevated the level of Aβ in culture medium significantly [24]. Our previous study has reported that repeated hypoxia treatment could lead to more and larger senile plaque formations and more Aβ42 production in aged APP^Swe^ + PS1^A246E^ double transgenic mice [25]. Interestingly, similar neuropathology was observed in the study of prenatal hypoxia in AD development in adult mice [26]. It has been found that prenatal chronic intermittent hypoxia significantly contributed to memory and cognition deficit in adult mice [26]. Moreover, prenatal hypoxia increased senile plaque formation and Aβ production in adult mice [26]. Another study also showed that hypoxia treatment impaired spatial learning and memory of AD transgenic mice, and caused ER stress and neuronal apoptosis [27]. These effects were associated with abnormal calpain activation because suppression of m-calpain expression could attenuate the hypoxia-induced ER stress and apoptosis [27].

### Chronic hypoxia may facilitate AD pathogenesis by increasing Aβ generation and decreasing Aβ degradation

Abnormal cleavaged β-amyloid peptide is the main constituent of neuritic plaques, which derives from sequential cleavage of APP by β-secretase and γ-secretase [28]. In the brain, the clearance of Aβ is mainly degraded by neprilysin (NEP) and insulin-degrading enzyme (IDE) [29]. Several studies including ours have shown that chronic hypoxia may elevate the production of Aβ and reduce the degradation of Aβ [25,26,30-33]. Chronic hypoxia in the human neuroblastoma SH-SY5Y cells caused reduced expression ADAM10, part of the α-secretases and increased the expression of β-site APP cleaving enzyme 1 (BACE1) [34,35]. Our previous study has demonstrated that chronic hypoxia increased Aβ generation by altering β- and γ-cleavage of APP [25]. The ratio of C99/C83 was elevated by chronic hypoxia in the brain of AD transgenic mice and the expression level of APH-1a was enhanced under hypoxia condition, which in turn would lead to increase in Aβ production [25]. Others have also shown that hypoxia could up-regulate BACE1 and APH-1a at both transcriptional and translational levels *in vitro* and *in vivo*[30,31,33]. It is widely accepted that hypoxia-inducible factor-1 (HIF-1) is the master regulator of the cellular response to chronic hypoxia [36]. Further investigations have revealed a functional hypoxia responsive element in the BACE1 gene promoter, to which HIF-1α can bind and result in increased activity of β-secretase under hypoxia conditions [30,33]. Similar HIF-1α binding site AP4 was identified in the promoter of APH-1a [31]. The binding of AP4 and HIF-1 to the promoter under hypoxia conditions may significantly affect the expression of APH-1a and lead to increased activity of γ-secretase [31]. All these findings suggest that hypoxia may increase the generation of Aβ *in vivo* and *in vitro*.

It is known that NEP plays a key role in the degradation of Aβ in the brain and it is one of the most important Aβ-degrading enzymes [29,37]. Evidence showed that NEP mRNA, protein and activity levels were declined not only in AD but also in the normal aging in the brains [38-42]. Fisk et al. demonstrated that hypoxia reduced NEP expression at the protein and mRNA levels as well as its activity [43,44]. We previously tested the expression levels of NEP in the cortex of AD transgenic mice, and found that the protein level of NEP started to reduce at the age of 6-month old in the brain of AD mice in comparison with age-matched wide-type mice [26]. Interestingly, the results also documented that chronic hypoxia induced significantly down regulation of NEP protein level in both transgenic mice and wide-type mice [26]. The declined level of NEP would reduce the clearance of Aβ. Given to the aggravated pathogenesis of AD and the over-load of Aβ burden under hypoxia condition, the chronic hypoxia-induced down regulation of NEP was implied to be a significant event in AD.

### Chronic hypoxia may aggravate Aβ burden through epigenetic modifications on genes associated with Aβ metabolism

**Figure 1:**
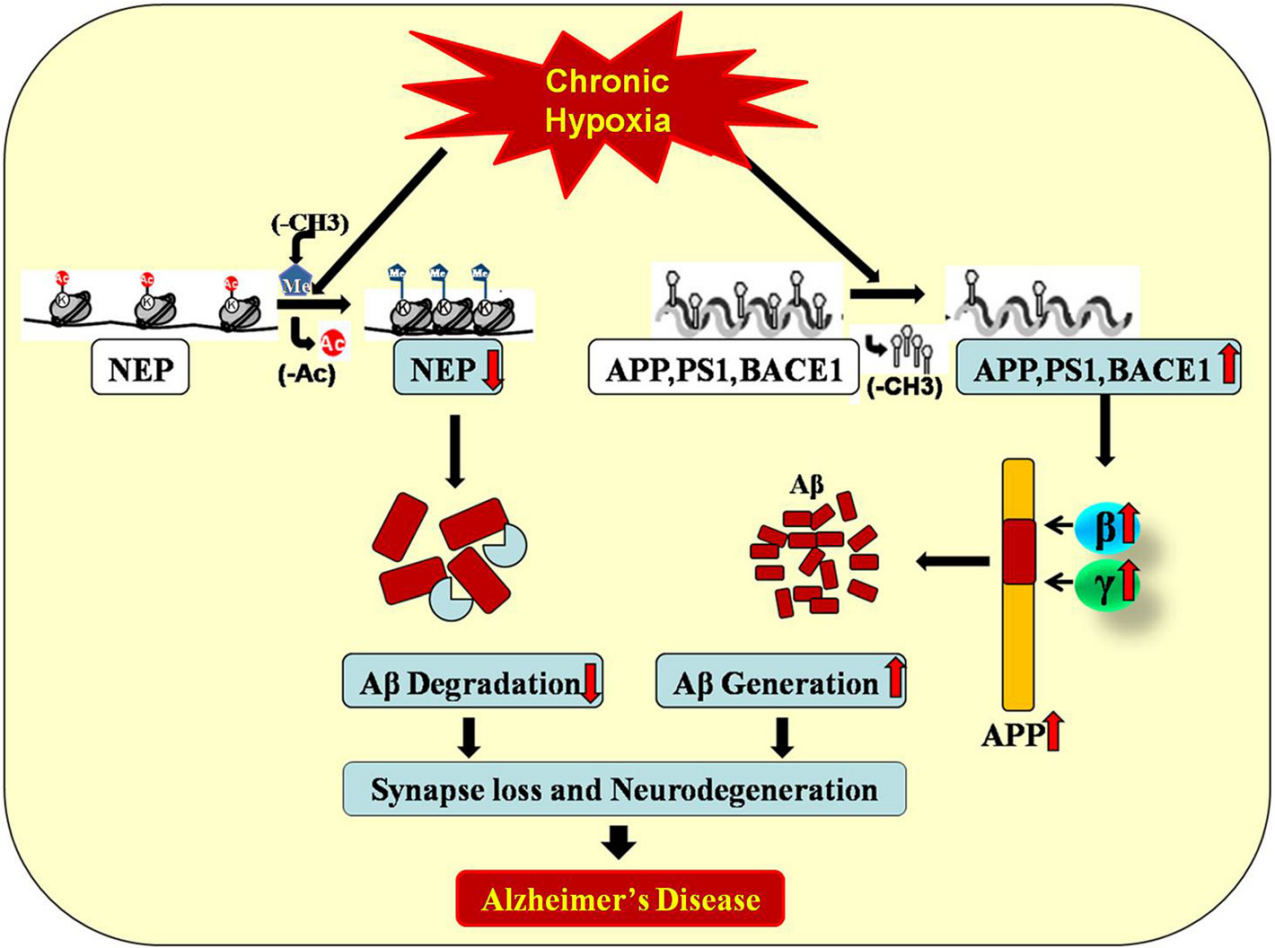
**The effect of chronic hypoxia on AD.** Hypoxia increases the β and γ cleavage of APP by demethylation in promoter CpG islands of APP, PS1, and BACE1. In addition, chronic hypoxia reduces the expression of NEP by up-regulating histone methyltransferase and histone deacetylase. All these contribute to the deposition of Aβ and AD pathogenesis.

It has been indicated that environmental factors and epigenetic mechanisms are likely to contribute to the etiology of LOAD [8-10]. Epigenetic mechanisms modify heritable and non-heritable traits without altering the underlying DNA code, mediated through the reversible modifications of DNA and histones [45]. Epigenetic processes play an important role in normal physical functions of cells and the body, so aberrant epigenetic modification are hypothesized to contribute to a majority of pathologies [46]. Abnormal PS1 methylation patterns have previously been associated with hypomethylation in promoter [47]. DNA demethylation has a strong correlation with transcriptional activation [48]. Hypomethylation in promoter CpG islands of other AD-associated genes such as APP and BACE1 has also been reported, which in turn may lead to abnormal up regulation of these genes and over-production of Aβ [49,50]. In the study of epigenetic differences in monozygotic twins discordant for AD, a significantly reduction of DNA methylation was observed in the temporal cortex neuronal nuclei of AD twin [51]. It was shown that hypoxia can reduce global DNA methylation in cancer cell lines *in vivo* and *in vitro*[52]. Importantly, hypoxia could cause long-lasting change in DNA methylation change in promoter regions, some of which could be highly correlated with transcriptional modulation in a number of genes involved in neural growth and development [53]. Chen et al. found that Aβ could reduce global DNA methylation and increase NEP promoter methylation and further suppress the NEP expression in mRNA and protein levels [54]. However, the methylation status of the NEP promoters did not regulate its expression *in vitro*[55], and chronic hypoxia did not affect the methylation patterns of NEP gene promoters in mouse primary cortical and hippocampal neurons [32]. Interestingly, chronic hypoxia could cause significant down regulation of NEP by up-regulating G9a histone methyltransferase and histone deacetylase 1 (HDAC1), which resulted in increased expression of H3K9me2 and decreased expression of H3-Ace respectively [32]. In addition, methylation inhibitor 5-Aza, HDAC inhibitor valproic acid VA, and siRNA-mediated knockdown of G9a or HDAC1 could reverse the expression of NEP [32]. Others also reported that the NEP promoter could be repressed by HDACs and the expression of NEP was repressed in neuronal cells via the competitive binding of HDACs to its promoter [55]. All these findings suggest that chronic hypoxia aggravates AD by epigenetic modulations, mainly focusing on DNA methylaion and histones modifications, which are of tremendous importance to the onset and progression of the disease (Figure 1).

### Epigenetic regulation targeting Aβ burden may help relieve the chronic hypoxia-mediated disease process

Currently, the treatment of AD focuses mainly on improving symptoms, targeting cholinergic and glutamatergic neuron transmission, with no therapeutics that can cure the disease [56]. Researchers around the world have worked hard to search for novel therapeutics for AD, which include the anti-Aβ (drugs to reduce its production, prevent its aggregation and promote its clearance), anti-tau (drugs to prevent its aggregation or phosphorylation), neurotrophins, and others [56]. Epigenetic regulation is a strong candidate therapeutic, since epigenetic modifications are reversible while genetic mutations are not. Drugs which can modulate DNA methylation and remodel the structure of chromatin through post-translational modifications of histones are promising potential candidates [57-60]. Recently, several drugs have been reported to be effective on AD mice models by modulating DNA methylation and histone acetylation separately, although none have yet entered clinical development [58,59,61,62]. Sodium valproic acid is one of these disease-modifying drugs, which may not only attenuate AD pathogenesis in transgenic AD mice, but also show effect in hypoxia-induced AD in the model. Valproic acid is a widely used antiepileptic drug, which has recently been found to have neuroprotection [63,64], and histone deacetylase inhibitory property may offer potential therapeutic option for AD [65]. It has been reported that valproic acid could inhibit Aβ production, reduce neuritic plaque formation, and improve behavioral deficits in AD mice [66]. Further study has demonstrated that valproic acid could attenuate the prenatal hypoxia-induced AD neuropathology, improved learning deficits and decrease Aβ42 levels in AD transgenic mice by up regulation of NEP [67]. Moreover, valproic acid could also restore memory deficit in adult offspring caused by prenatal hypoxia via elevating NEP expression and activity [68]. Hence, epigenetic drugs that increase histone acetylation by inhibiting HDAC could be useful in the disease treatment. Strategies targeting Aβ burden by epigenetic regulations, may represent new opportunities for therapeutic intervention to AD.

## Conclusion

Epigenetic effects can present throughout life and environmental factors can influence the individuals repeatedly along their whole life. A wide range of clinical diseases may be associated with chronic hypoxia including obstructive sleep apnea, cerebrovascular diseases, systemic hypertension, cardiovascular disease, chronic obstructive pulmonary disease, pulmonary hypertension, congestive heart failure and others [69]. Given to the possibility that chronic hypoxia is associated with AD, more attention should be paid to this pathological event and the underlying mechanisms. In addition, we hypothesize that prevent chronic hypoxic condition may be helpful for the reduction of AD. Unlike genetic mutations in FAD, epigenetic alterations are reversible, which are easier to modulate the process. Therefore, understanding the mechanisms of epigenetic alteration during AD pathogenesis caused by environmental factors, is essential for searching new treatment strategy for the disease.

## Abbreviations

AD: Alzheimer’s disease; NFTs: Neurofibrillary tangles; Aβ: beta-amyloid; FAD: Familial AD; LOAD: Late-onset form of AD; APP: Amyloid-precursor protein; PS: Presenilin; Apo E: Apolipoprotein E; GWAS: Genome-wide association studies; CLU: Clusterin; PICALM: Phosphatidylinositol binding clathrin assembly protein; CR1: Complement receptor 1; BIN1: Bridging integrator; MARK4: Microtubule affinity-regulating kinase4; ABCA7: The ATP cassette transporter; MS4A6A: A membrane-spanning 4-domains, subfamily A; CD2AP: A CD2-associated protein; EPHA1: Ephrin A1; Health ABC: Health, Aging, and Body Composition; NEP: Neprilysin; IDE: Insulin-degrading enzyme; HIF-1: Hypoxia-inducible factor-1; BACE: Beta-site APP-cleaving enzyme; CBZ: Carbamazepine.

## Competing interests

All authors declare no competing financial interests.

## Authors’ contributions

HL reviewed the literature and has written the initial manuscript draft; WL reviewed and critiqued the manuscript. Both authors read and approved the final manuscript.
